# A Vulnerability Index to Assess the Risk of SARS-CoV-2-Related Hospitalization/Death: Urgent Need for an Update after Diffusion of Anti-COVID Vaccines

**DOI:** 10.3390/idr16020021

**Published:** 2024-03-15

**Authors:** Francesco Lapi, Ettore Marconi, Alexander Domnich, Iacopo Cricelli, Alessandro Rossi, Ignazio Grattagliano, Giancarlo Icardi, Claudio Cricelli

**Affiliations:** 1 Health Search, Italian College of General Practitioners and Primary Care, 50142 Florence, Italy; 2 Hygiene Unit, San Martino Policlinico Hospital-IRCCS for Oncology and Neurosciences, 16132 Genoa, Italy; alexander.domnich@hsanmartino.it (A.D.); icardi@unige.it (G.I.); 3 Genomedics S.r.l., 50141 Firenze, Italy; iacopo.cricelli@genomedics.it; 4 Italian College of General Practitioners and Primary Care, 50142 Florence, Italy; rossi.alessandro@simg.it (A.R.); studiomedico@grattagliano.it (I.G.); cricelli@gmail.com (C.C.); 5 Department of Health Sciences, University of Genoa, 16132 Genoa, Italy

**Keywords:** COVID-19, death, hospitalization, primary health care, prediction model, vaccination

## Abstract

**Background**: There are algorithms to predict the risk of SARS-CoV-2-related complications. Given the spread of anti-COVID vaccination, which sensibly modified the burden of risk of the infection, these tools need to be re-calibrated. Therefore, we updated our vulnerability index, namely, the Health Search (HS)-CoVulnerabiltyIndex (VI)d (HS-CoVId), to predict the risk of SARS-CoV-2-related hospitalization/death in the primary care setting. **Methods**: We formed a cohort of individuals aged ≥15 years and diagnosed with COVID-19 between 1 January and 31 December 2021 in the HSD. The date of COVID-19 diagnosis was the study index date. These patients were eligible if they had received an anti-COVID vaccine at least 15 days before the index date. Patients were followed up from the index date until one of the following events, whichever came first: COVID-19-related hospitalization/death (event date), end of registration with their GPs, and end of the study period (31 December 2022). To calculate the incidence rate of COVID-19-related hospitalization/death, a patient-specific score was derived through linear combination of the coefficients stemming from a multivariate Cox regression model. Its prediction performance was evaluated by obtaining explained variation, discrimination, and calibration measures. **Results**: We identified 2192 patients who had received an anti-COVID vaccine from 1 January to 31 December 2021. With this cohort, we re-calibrated the HS-CoVId by calculating optimism-corrected pseudo-R^2^, AUC, and calibration slope. The final model reported a good predictive performance by explaining 58% (95% CI: 48–71%) of variation in the occurrence of hospitalizations/deaths, the AUC was 83 (95% CI: 77–93%), and the calibration slope did not reject the equivalence hypothesis (*p*-value = 0.904). **Conclusions**: Two versions of HS-CoVId need to be differentially adopted to assess the risk of COVID-19-related complications among vaccinated and unvaccinated subjects. Therefore, this functionality should be operationalized in related patient- and population-based informatic tools intended for general practitioners.

## 1. Background

To categorize the risk of clinical progression of SARS-CoV-2 infection and to assess the risk of COVID-19-related complications, we recently developed and validated a score (i.e., hospitalization/death), namely, the Health Search (HS)-CoVulnerabiltyIndex (VI)d [1]. It combines regulatory recommendations [2] with other clinical and demographic information registered in electronic health records (EHRs). Such a tool, which fairly predicts (pseudo-R^2^ = 60%; AUC = 80%; and slope (1.01; *p* < 0.770) and intercept (0.12; *p* < 0.169) calibration) COVID-19-related complications for a 30-month event horizon, empowers General Practitioners (GPs) to prioritize the most susceptible patients to receive the initial anti-COVID vaccine or any subsequent booster doses. Before the development of the HS-CoVId, similar tools were available in Italy and other countries, but they were intended for use in hospital/specialist settings and/or tailored to clinicians with a particular expertise [3,4,5]. On the other hand, there was no similar score for primary care physicians, whose patients’ datasets are certainly characterized by greater dimensions and major heterogeneity.

This tool, once implemented in software intended for GPs, besides being country-specific must be compliant with local regulatory and policy decisions. The HS-CoVId is mainly based on these criteria along with other clinical information embedded in EHRs. Prior COVID-19 infections and profession-related categories are tailoring factors which have to be accounted for by GPs as well. It is therefore crucial for GPs to verify the placement of their individual patients in the related risk stratum and compare them with other patients classified into different subgroups, thus allowing the physicians to prioritize certain treatment and hospitalization in positive patients and vaccination in those at high-risk for respiratory complications but who are still uninfected.

**Figure 1:**
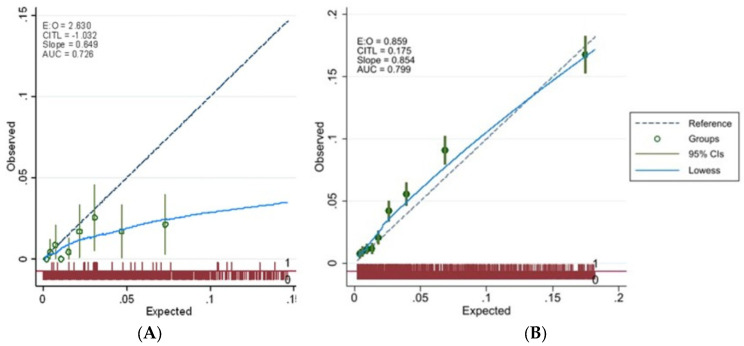
Calibration plot showing expected vs. observed risk of COVID-19-related hospitalization/death over 1-month follow-up, according to the HS-CoVId in vaccinated (**A**) and unvaccinated (**B**) subjects. LOWESS (Locally Weighted Scatterplot Smoothing).

For these reasons, each prediction score needs to be regularly updated, given the overtime changes of biological, therapeutic, and environmental factors which could lead to algorithm mis-calibration [6]. The speed of this update strictly depends on disease evolution which, in the case of SARS-CoV-2, as with other infectious diseases, might even be performed on a daily basis. Indeed, the HS-CoVId was based on data stemming from pre-vaccine era, and the sensible reduction in hospitalization rates and fatal events among vaccinees [7,8] made us to question whether the score was currently mis-calibrated for risk prediction. As a matter of fact, when we attempted to apply the individual score to those who were immunized with an anti-COVID vaccine in 2021 (i.e., the Italian GPs were actively involved in the vaccination campaign by administering vaccines in their own clinics and/or in vaccination hubs), its predictive ability sensibly differed between vaccinated and unvaccinated subjects. As shown in Figure 1, while the HS-CoVId still performs well in unvaccinated subjects, there is a clear overestimation of risk among immunized subjects. This evidence is largely consistent with findings on the effectiveness of anti-COVID vaccines, along with available studies on their favorable safety profiles [9].

As in several countries, GPs have a key role in influenza vaccination campaigns, which has been recently “re-defined” as influenza-anti-COVID campaigns by the healthcare authorities [8]. They are indeed providing GPs with further doses of anti-COVID vaccines for older and at-risk subjects to be co-administered with flu vaccination. The HS-CoVId re-calibration was therefore needed to provide GPs with a reliable tool to prioritize anti-COVID vaccines and/or its booster doses.

## 2. Methods

### 2.1. Data Source

The Health Search Database (HSD) is a comprehensive, longitudinal database that contains electronic health records (EHRs) of roughly 1 million adults. The HSD was established in 1996 by the Italian College of General Practitioners and Primary Care, with the aim to conduct clinical research in the primary care setting. This database includes demographic and clinical data, which are linked through a unique encrypted code that tracks various aspects of a patient’s health, such as drug prescriptions, lifestyle factors, clinical investigations, hospitalizations, and deaths. The diagnoses and prescribed medications are coded using the International Classification of Diseases, 9th Revision, Clinical Modification (ICD-9-CM) and Anatomical Therapeutic Chemical (ATC) systems, respectively. The other variables are registered using regional coding systems. The study included 800 general practitioners who met the quality criteria and served approximately 1.2 million patients. These GPs, homogenously distributed across Italy, attended specific courses for data entry according to the HSD methodology for data collection. The HSD is currently listed in the EMA RWD official catalog [10] and it has been previously adopted for epidemiological research including the development and validation of prediction scores [1,11,12].

### 2.2. Study Design and Data Analyses

To re-calibrate the HS-CoVId, we adopted the same methodology leading to the previous version of the score, which is described elsewhere [1]. This analysis was compliant with the TRIPOD statements [13]. In brief, we formed a cohort of individuals aged ≥15 years and diagnosed with COVID-19 (ICD-9-CM: 460/30; 480.9/60) by any laboratory method or clinically between 1 January and 31 December 2021 in the HSD. The date of COVID-19 diagnosis was the study index date. These subjects were eligible if they had received an anti-COVID vaccine at least 15 days before the index date. Those younger than 15 years old and with no registered anti-COVID vaccination in the 15 days preceding COVID-19 diagnosis were excluded. The selected individuals were monitored from the index date until the earliest of the following events occurred: hospitalization or death related to COVID-19 (event date), discontinuation of their registration with their GPs, or the end of the study period on 31 December 2022. For the purpose of this study, COVID-19-related hospitalizations were defined as those in which the terms “SARS-CoV-2”, “COVID*”, or “coron*” were reported in the code description field and the hospitalization took place in departments such as “intensive”, “respiratory”, or “emergency”. Fatal cases were those occurring within 30 days of the diagnosis of SARS-CoV-2 infection. In line with the prior study, each record was then manually reviewed by an expert clinician to verify the veracity of the event definition [1]. All available demographic and clinical determinants forming the score stemmed from systematic evidence [14,15] and official documents issued by the Italian Health Authorities [8]; every covariate is operationally described in the primary study on the creation of the HS-CoVId [1].

Descriptive statistics were presented in the form of means with standard deviations (SDs) and proportions with 95% confidence intervals (CIs) for continuous and categorical variables, respectively. The incidence rate of COVID-19-related hospitalizations and deaths was calculated by dividing the number of events by the person-months accumulated during the follow-up period. A multivariate Cox regression model was used to estimate the regression coefficients for each covariate, with the effect size expressed as an adjusted hazard ratio (aHR) along with a 95% CI. A patient-specific score was derived by combining these coefficients linearly, resulting in the HS-CoVId score. The 30-day predicted risk of experiencing the study outcomes was calculated as a function of the cumulative baseline hazard and the linear predictor, which is the sum of the product of the predictor values for the individual patient and the beta coefficients for each risk factor. To validate the score, we calculated pseudo-R2 and AUC as overall performance and discrimination measures, respectively. We also calibrated the score by comparing the predicted vs. observed risks of COVID-19-related hospitalizations and deaths. We provided optimism-corrected pseudo-R2, AUC, and calibration slope by bootstrapping 200 random samples from the study cohort [6,16]. To make the score more easily interpretable and applicable for general practitioners, we categorized it into different subgroups, including a low/intermediate-risk category and a high-risk category, using cut-off points based on the predicted risk of COVID-19-related complications as determined by Cox’s methods [14].

Given the reduced size of the study cohort, we conducted a secondary analysis to verify the robustness of the results. We ran the primary model again in the entire cohort of vaccinees (*N* = 124,320) irrespective of the COVID-19 diagnosis. By doing so, we were able to identify more cases of hospitalizations/deaths, thereby ensuring the analysis power among immunized patients. For this analysis, we re-calculated pseudo-R^2^ and AUC as performance and discrimination measures, respectively. In addition, we determined the sensitivity and specificity with a precise level of uncertainty, utilizing our increased analytical power. The calibration slope and optimism-corrected measures were re-calculated as well [6,16]. We adopted the same selection criteria to form another validation cohort using the 2023 update (i.e., up to June 2023) of the HSD. By doing so, a temporal validation of the algorithm was performed as a proxy of the external validation test [6].

The scientific Internal Review Board of the Italian College of General Practitioners and Primary Care approved this study.

## 3. Results

A total of 2192 patients were included in the study, with 51.7% of them being female and a mean age of 52.3 years (SD 18.4). During the follow-up period, 57 cases of COVID-19-related hospitalizations and/or deaths were recorded, resulting in an overall incidence rate of 1.4 per 100 person-years (95% CI: 1.1–1.8). The Cox model revealed coefficients related to age, sex, and clinical characteristics that pertained to highly vulnerable and/or severely disabled patients, as per regulatory indications, and/or those with other vulnerabilities, such as other risk factors like diabetes with no complications and hypertension, coded in the EHRs. Among vaccinees, they were linearly combined to form the HS-CoVId, which was then categorized in deciles in order to evaluate its prediction accuracy.

The model explained 62% (95% CI: 55–78%) of variance in the occurrence of hospitalizations/deaths; the discrimination accuracy was higher than 80% (AUC 82% (95% CI: 75–79%)); in terms of calibration, over a 30-day follow-up, both calibration intercept (0.3 (95% CI: −0.25–0.83)) and slope (1.00 (95% CI: 0.81–1.20)) did not reject the equivalence hypothesis (*p*-value equal to 0.951 and 0.286, respectively). When the explained variance, discrimination and calibration measures, and related 95% CI were calculated after bootstrapping 200 samples, the results were consistent with those reported above (Table 1). Along this line, the secondary analysis on the entire cohort of 124,320 vaccinees with 2123 events (incidence rate equal to 1.9 (95% CI: 1.8–2.0) person-years) provided results which were largely consistent with those described above. The model explained 48% (95% CI: 46–50%) of variance in the occurrence of hospitalizations/deaths; the discrimination accuracy was higher than 80% (AUC 88% (95% CI: 87–90%)); in terms of calibration, over a 30-day follow-up, the calibration slope (1.00 (95% CI: 1.00–1.04)) did not reject the equivalence hypothesis (*p*-value equal to 0.436). Similar findings were obtained for the optimism-corrected estimates after bootstrapping (Table 2).

According to the Cox methodology, we identified low/moderate-risk (*n* = 90,743 (73%)) and high-risk (*n* = 33,577 (27%)) categories, with a sensitivity and specificity of 92% (95% CI: 88–95%) and 70% (95% CI: 69–70%), respectively. In the first semester of 2023 (*N* = 126,161; mean age: 61 (SD: 19.1), 54.1% female), 35,269 (28.0%) and 90,892 (72%) patients were classified as low/moderate and high risk, respectively. The related sensitivity, specificity, pseudo-R^2^, and AUC were 68% (95% CI: 68–70%), 87% (95% CI: 86–89%), 90.0% (95% CI: 86.2–93%), and 67.6% (95% CI: 67.3–68.0%), respectively.

## 4. Discussion

With this analysis, we updated the HS-CoVId which was previously developed and validated in 2020 during the pre-vaccine era. The score was tested for its prediction accuracy among patients who had received an anti-COVID vaccine in 2021, and all validation measures were consistent with those calculated with the primary version of the score [1]. As such, these findings confirm the need for a score and its regular update to predict the risk of COVID-19-related complications in primary care. Indeed, while similar evidence-based tools were available in the medical literature, they were based on in-hospital settings [3,4] or were not trained in actual COVID-19 infections identified early by GPs [5]. This update, in addition to providing further evidence of anti-COVID vaccine effectiveness (i.e., observed out of predicted risks of COVID-19-related hospitalization/death were sensibly reduced) [17,18], shows that HS-CoVId-based estimation of risk is currently able to differentiate between vaccinated and unvaccinated subjects.

Three other prognostic scores for severe respiratory failure have been developed in Italy [3,4,19]. The PREDI-CO score, developed on 1113 in-patients, demonstrated excellent discrimination ability (AUC 0.89) in predicting severe respiratory failure by combining clinical information and laboratory findings [4]. The Brixia score, designed for experienced radiologists, showed good agreement (κ 0.82) between chest X-ray findings and severe in-hospital progression of COVID-19 [19]. Cecconi et al. [3] provided a reliable (C 0.845) tool to predict the risk of clinical deterioration in hospitalized patients using respiratory rate, blood gas parameters, history of coronary heart disease, C-reactive protein, and serum creatinine levels.

Nevertheless, these scores did not meet general practice’s needs, given the required determinants to retrieve an estimation along with the wider clinical heterogeneity of patients being cared for by GPs. In this context, practical implementations of our results in general practice are even more crucial. In GPs’ informatic tools, the score might be automatically provided by combining demographic and clinical features according to the pre-established programmed criteria. The HS-CoVId would allow the view of two different individualized scores whether anti-COVID-19 vaccination is present or not. Specifically, two main implementations of the updated version of the HS-CoVId should be considered. First, the HS-CoVId might be operationalized into a Clinical Decision Support System (CDSS), with which GPs can visualize a patient-based dashboard indicating the individual risk. This approach can easily be implemented in primary care settings, as electronic medical charts are currently widely available and mandatory for GPs to use when providing care (D.M. 4 March 2009 [G.U. n. 146 del 26 June 2009]; DPCM March 26, 2008 [G.U. 28 May 2008, n. 124]). Additionally, according to a population-based approach, GPs can create a list of “high-risk” patients for whom they can plan immunization schedules and/or explore other treatment options. For example, GPs may choose to contact patients who do not schedule vaccination appointments (primary or booster injections) spontaneously based on their individual HS-CoVId score.

In Italy, this algorithm might support the GPs during the next influenza vaccination campaign in which the public health authority recommends the co-administration of influenza and COVID vaccines for those at risk (as per the combination of risk factors for progression of the infectious disease) of developing respiratory-related complications. On average, Italian GPs have 10.3 encounters with patients per year. The majority of these encounters are due to older adults, especially those aged 85 and above, who may have up to 20 or more encounters per patient/year. According to the data from the Health Search reports [20], a GP with a maximum of 1500 patients would expect to have 420 patients classified as high-risk vaccinees, requiring frequent evaluations. Considering the importance of vaccination campaigns for the National Health Service (NHS), the expected workload from these decision tools should be deemed acceptable and cost-effective for both GPs and the NHS.

The combination of patient- and population-based tools embedding the HS-CoVId might therefore effectively optimize the immunization procedures, given their proven need in clinical decision-making, especially in case of reduced availability of vaccines, delay of vaccine deliveries, vaccine hesitancy in at-risk populations, and/or issues in organization of primary care clinics and their interaction with in-hospital departments for vaccination. The population-based tool performing on the EHR-based HS-CoVId would automatically stratify patients according to their vulnerability; within those classified as highly vulnerable in case of a contagion, further staging might be obtained for those aged 60 years or older, on their level of frailty levels as assessed by our Primary Care Frailty Index [21].

Furthermore, such a system would sustain GPs to participate in networks and create a real-time epidemiological observatory. Through these data, GPs and public health authorities would be able to plan, monitor, and implement prevention strategies, given the absence of epidemiologic observatory for most vaccinations in adults.

This study has some limitations. First, the fact that the multivariate model was based on a few cases of COVID-19-related hospitalizations/deaths (*n* = 57) provided some unstable estimates. For this reason, we validated the score using bootstrapping instead of cohort split-samples [22,23], as well as performed a secondary analysis on the entire cohort of vaccinees irrespective of the COVID-19 diagnosis. Reassuringly, the results stemming from bootstrapping and the secondary analysis were always consistent with those reported for the primary analysis. Second, we were unable to validate the score using an external (independent) population. Reassuringly, the prediction accuracy of the score was largely consistent with its previous version, which was externally validated [1]; similar findings were gathered when the algorithm was applied to the first semester of 2023 on a temporal validation cohort. That being said, when these algorithms are developed through a representative data source with the aim to apply this score in the same setting, the internal validity is sufficient [24]. In this respect, the HS-CoVId might be adopted in similar settings (e.g., countries with similar primary care role), but its re-calibration (i.e., external validation) would be necessary to demonstrate the prediction accuracy in these different populations. Third, given that vaccination hubs were largely involved in the first phase of the vaccination campaign, GPs could have under-registered some injections, thus increasing the presence of false-negatives for vaccinees. Reassuringly, the level of sensitivity of vaccine exposure was sufficient to obtain different calibration among vaccinees.

In Italy, GPs are actively included in the anti-COVID vaccination campaign and will soon cover further key roles to fight the pandemic and its current transition to an endemic phase. The use of anti-COVID vaccines is moving toward a personalized approach [25] with major focus and prioritization on most vulnerable and/or frail patients. The use of vaccines, and their updated versions against new variants, should be therefore tailored on individual subjects. To do so, GPs need to be equipped with regularly updated evidence-based scoring systems embedded in their informatic tools.

## Figures and Tables

**Table 1:** Measurements of prediction accuracy for the HS-CoVId with a 30-day follow-up for the overall cohort and after bootstrapping.

	Overall Cohort (*N* = 2195)	Bootstrapping (*n* = 200 Samples)
Explained variation		
Pseudo-R^2^	0.619 (0.555–0.767)	0.575 (0.474–0.711)
Discrimination		
AUC	0.823 (0.751–0.893)	0.829 (0.770–0.930)
Calibration		
Slope	1.000 (0.805–1.195)	1.010 (0.800–1.179)
*p*-value	0.9507	0.9406
Intercept	0.293 (−0.246–0.832)	-
*p*-value	0.2863	-

**Table 2:** Measurements of prediction accuracy for the HS-CoVId with a 30-day follow-up for the cohort of vaccinees and after bootstrapping.

	Overall Cohort (*N* = 124,320)	Bootstrapping (*n* = 200 Samples)
Explained variation		
Pseudo-R^2^	0.478 (0.460–0.504)	0.467 (0.445–0.492)
Discrimination		
AUC	0.883 (0.869–0.897)	0.889 (0.874–0.905)
Calibration		
Slope	1.000 (0.962–1.038)	0.996 (0.948–1.040)
*p*-value	0.436	

## Data Availability

Data are contained within the article.
